# Over-expression of miR-223 induces M2 macrophage through glycolysis alteration and attenuates LPS-induced sepsis mouse model, the cell-based therapy in sepsis

**DOI:** 10.1371/journal.pone.0236038

**Published:** 2020-07-13

**Authors:** Cong Phi Dang, Asada Leelahavanichkul

**Affiliations:** 1 Medical Microbiology, Interdisciplinary and International Program, Graduate School, Chulalongkorn University, Bangkok, Thailand; 2 Department of Microbiology, Faculty of Medicine, Chulalongkorn University, Bangkok, Thailand; 3 Translational Research in Inflammation and Immunology Research Unit (TRIRU), Department of Microbiology, Chulalongkorn University, Bangkok, Thailand; National Institutes of Health, UNITED STATES

## Abstract

The attenuation of hyper-inflammation in sepsis with the administration of anti-inflammatory macrophages is an interesting adjuvant therapy for sepsis. Because the induction of anti-inflammatory macrophages by microRNA (miR), a regulator of mRNA, has been mentioned, the exploration on miR-induced anti-inflammatory macrophages was performed. The over-expression of miR-223 and miR-146a in RAW264.7 induced M2 macrophage-polarization (anti-inflammatory macrophages) as evaluated by the enhanced expression of *Arginase-1* and *Fizz*. However, miR-223 over-expressed cells demonstrated the more potent anti-inflammatory property against LPS stimulation as lesser *iNOS* expression, lower supernatant IL-6 and higher supernatant IL-10 compared with miR-146a over-expressed cells. Interestingly, LPS stimulation in miR-223 over-expressed cells, compared with LPS-stimulated control cells, demonstrated lower activity of glycolysis pathway and higher mitochondrial respiration, as evaluated by the extracellular flux analysis, and also down-regulated *HIF-1α*, an important enzyme of glycolysis pathway. In addition, the administration of miR-223 over-expressed macrophages with IL-4 pre-conditioning, but not IL-4 stimulated control cells, attenuated sepsis severity in LPS injected mice as evaluated by serum creatinine, liver enzymes, lung histology and serum cytokines. In conclusion, miR-223 interfered with the glycolysis pathway through the down-regulation of *HIF-1α*, resulting in the anti-inflammatory status. The over-expression of miR-223 in macrophages prevented the conversion into M1 macrophage polarization after LPS stimulation. The administration of miR-223 over-expressed macrophages, with IL-4 preconditioning, attenuated sepsis severity in LPS model. Hence, a proof of concept in the induction of anti-inflammatory macrophages through the cell-energy interference for sepsis treatment was proposed as a basis of cell-based therapy in sepsis.

## Introduction

Sepsis, a syndrome of organ dysfunction due to the dys-regulation of host responses to systemic infection, is a major cause of death in patients with clinically ill conditions and has been recognized as an important world-wide healthcare problem [1]. The imbalance of pro- and anti-inflammatory immune-responses is one of the important causes of death in sepsis and the anti-inflammation is an interesting adjunctive treatment of sepsis, especially during the pro-inflammatory state [2–4]. Indeed, compelling evidences indicate that robust innate immune responses, including macrophages, mainly accounts for sepsis pathophysiology [5].

Macrophages, heterogeneous immune cells with pleiotropic functions [6], are fundamentally divided into classically M1 and alternative M2 polarization with pro- and anti-inflammatory property, respectively [7]. Interestingly, macrophages in sepsis hyper-inflammation are predominant in M1 polarization and are responsible for the production of inflammatory mediators [8]. As such, the induction or the administration of anti-inflammatory M2 macrophages during sepsis hyper-inflammation is mentioned [9, 10]. In addition, microRNA (miR), a small non-coding single-strand RNA [11], regulates post-transcriptional messenger RNA (mRNA) and inhibits protein-translation [12]. Because one miR is able to silence multiple targets that are responsible for numerous biological processes [13], the manipulation of macrophage-polarization with miR is interesting [14]. Accordingly, miR-223 is essential for M2 polarization [15] and the down-regulation of miR-223 in patients with severe sepsis possibly induces predominant M1 polarization [16]. In parallel, miR-146a is well documented as an anti-inflammatory miR, preferentially augments M2 polarization [17], that attenuates sepsis-induced cardiac injury in a mouse model [18].

Interestingly, cellular metabolism is essential for immune cells [19], including macrophages [20], as the metabolic signatures of M1 and M2 macrophage-polarization are glycolysis and mitochondrial respiration, respectively [21]. Indeed, the induction of macrophage plasticity through the interference of cell energy and cell metabolism by miRs is mentioned as miR-33 augmented glycolysis and induced M1 macrophage-polarization in an atherosclerosis mouse model [22]. Although the impact of miRs upon malignant-cell metabolism is well-known [23], the information about influence of immune cells metabolism upon sepsis is still very little. Moreover, the anti-inflammatory potency of several miRs might be different, for example, miR-223 mediated anti-inflammation through HIF-1α, PPARγ and STAT3 [15, 24–26], while miR-146a inhibits IRAK1 and TRAF6 [27]. Here, the candidate miRs were over-expressed in macrophages and examined several cell characteristics, including cell energy, before using as a cell-based therapy in a sepsis LPS injection mouse model.

## Materials and methods

### Animal and animal model

Animal care and use protocol are based upon the National Institutes of Health (NIH), USA. The protocol was approved by the Institutional Animal Care and Use Committee of the Faculty of Medicine, Chulalongkorn University, Bangkok, Thailand. Male 8-wk-old mice on C57BL/6 background were purchased from Nomura Siam International (Pathumwan, Bangkok, Thailand) Mice were at rest for 1 wk in the animal facility before use and endotoxin (LPS) injection model was performed following previous publications [28–30]. Briefly, 4 mg/kg of endotoxin (LPS) from *Escherichia coli* 026:B6 (Sigma-Aldrich, St. Louis, MO, USA) was administered intra-peritonium (ip) at 1 h after the tail vein injection by miR over-expressed RAW264.7 preconditioning with IL-4 (detail later) at 1x10^6^ CFU diluted in 0.3 ml phosphate buffer solution (PBS) as previously described [31–34]. Blood collection through tail vein was performed at 3 days before LPS-injection (0 h) and at 2 and 4 h post-LPS. Mice were sacrificed at 6 h post LPS-injection by cardiac puncture under isoflurane anesthesia. Of note, some mice were sacrificed at these time-points for organ collection. Serum creatinine (Cr) by QuantiChrom Creatinine-Assay (DICT-500, BioAssay, Hayward, CA, USA), serum aspartate transaminase (AST) by EnzyChrom AST assay (EASTR-100, BioAssay), serum alanine transaminase (ALT) by EnzyChrom ALT assay (EALT-100, BioAssay) and serum cytokines by ELISA assays (ReproTech, Oldwick, NJ, USA) were analysed. In addition, blood leukocyte determination was performed as previous published [35]. In short, blood was mixed with 3% acetic acid, a hemolytic solution, with a ratio of blood: acetic acid at 6:100 by volume before counting with a hemocytometer. In parallel, Wright-stained blood smear was examined for the percentage of polymorphonuclear cells (PMN) and lymphocyte. The total number of these cells was calculated by the total count from hemocytometer multiplied by the percentage of cells from the Wright-stained slide.

### Cell tracker and histological analysis

Macrophages (RAW264.7) at 3x10^6^ cells per plate were incubated with cell-labeling Carboxy-fluorescein diacetate succinimidyl ester (CFDA-SE; Sigma-Aldrich), at 25 μM in PBS, for 30 minutes at 37°C according to the manufacturer’s instructions. Then the buffer was gently aspirated and further incubated with warm media for 15 minutes before collecting the cells. Passively-diffused CFDA-SE in cytoplasm is cleaved by intracellular-esterase to form carboxyfluorescein succinimidyl ester with the fluorescent activity. CFDA-SE—labeled cells are confirmed under fluorescent microscope before the intravenous (iv) injection with 1x10^6^ cells/ mouse. Then, mice were sacrificed with cardiac puncture under isoflurane anesthesia with organ collection in the optimal cutting temperature compound (OCT) at -80°C before cutting into 4 μm-thick slides and DAPI (4′, 6-diamidino-2-phenylindole) (Thermo fisher Scientific) was incubated for 10 minutes at room temperature for nuclear counter stain. Each slide was observed with fluorescein microscope at 10 random 200X fields for the enumeration of fluorescent-positive tracked-cells per field as the representatives for cell burden in organs.

For organ injury analysis, the organs were fixed in 10% formalin before Hematoxylin and Eosin (H&E) staining process and the semi-quantitative analysis of organ injury. As such, lung injury was analyzed in accordance with previous publications [36, 37] through evaluation of area of the injury as determined by alveolar hemorrhage, alveolar congestion, neutrophil infiltration and alveolar wall thickness. Semi-quantitative scores were given in each observed field at 200x magnifications according to the following: 0 points, no injury in the observed field; 1 point, injury up to 25%, 2 points, injury up to 50%; 3 points, injury up to 75%; 4 points, injury in the entire field. The scores from 20 fields of each slide were calculated and were presented as the representative lung injury parameter.

### Micro-RNA transfection and the preparation of IL-4 preconditioning cells

**Fig 1:**
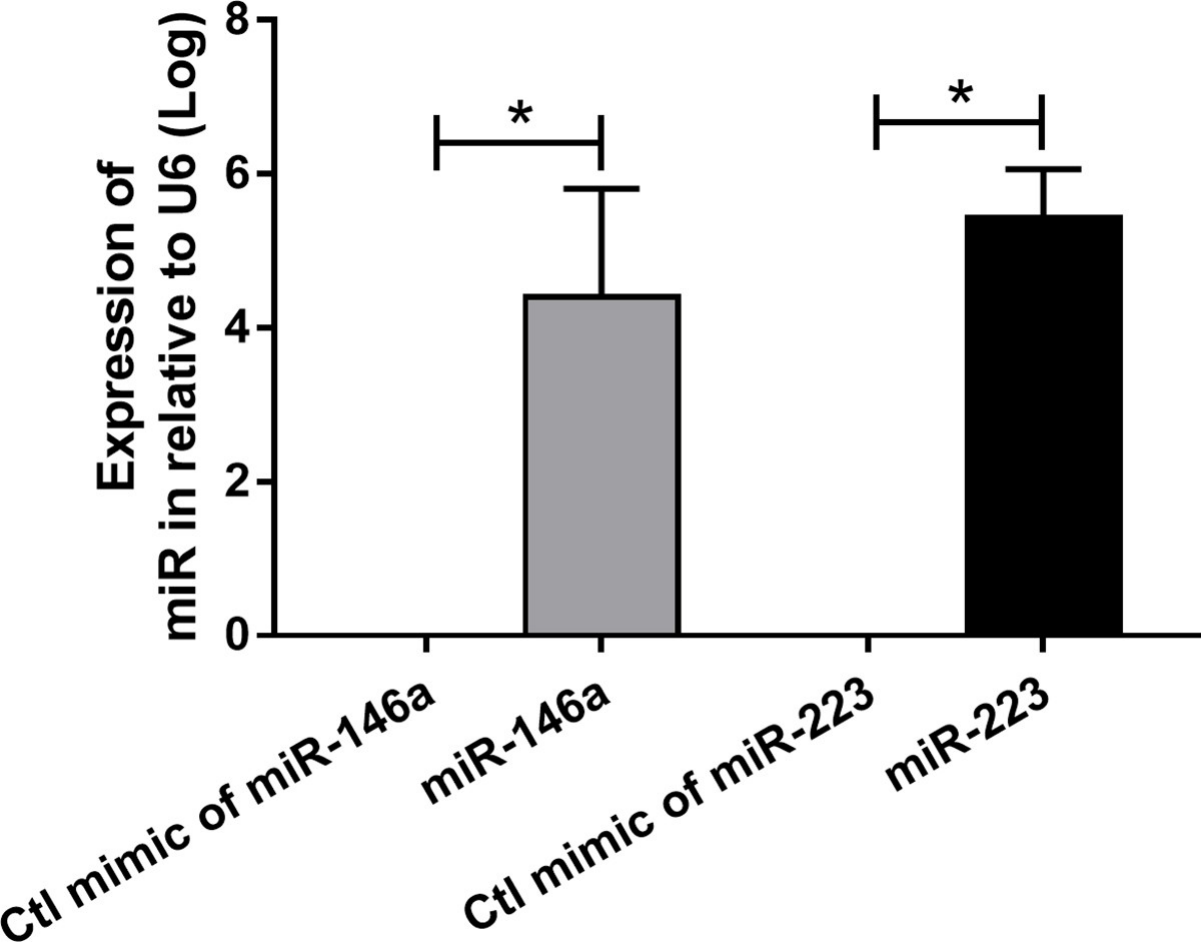
Transfection efficiency of cell culture. Transfection efficiency of RAW264.7 cell with miR-146a and miR-223 was determined by quantitative polymerase chain reaction using Taqman probe in relative to the expression of housekeeping gene U6.

Murine macrophage cell line (RAW264.7) were cultured in DMEM supplemented with 10% heat-inactivated fetal bovine serum (FBS) and Penicillin-Streptomycin (Thermo fisher Scientific, Waltham, MA, USA) in 5% carbon dioxide (CO_2_) at 37°C. Then the microRNA (miR) was transfected into 1.5x10^6^/ well of macrophages in 24-well plate following a previous protocol [38]. Briefly, the transfection mixture consisted of 5 pmol of miR (miR146a, miR223 or mimic control) (Ambion, Austin, TX, USA) and 1.5 μl Lipofectamin RNAimax reagent (Invitrogen, Carlsbad, CA, USA) in Opti-MEM (Gibco, Thermo Fisher Scientific) for one reaction was incubated with cells for 2 days before the verification of transfection-efficiency by quantitative polymerase chain reaction (qPCR) as demonstrated in Fig 1. In addition, M1 or M2 polarization was activated by LPS (10 ng/ml) or IL-4 (20 ng/ml), respectively, before cell collection and supernatant cytokines were measured by ELISA (ReproTech). For preparation of adoptive transfer, miR over-expressed RAW264.7 were treated with IL-4 (20 ng/ml) to activate M2 macrophage polarization, washed 2 times by PBS and intravenously administered into mice at 1 hour prior to LPS intra-peritoneal injection (4 mg/kg).

### The determination of macrophage polarization by quantitative Polymerase Chain Reaction (qPCR)

The qPCR was performed following previous publications [29]. Briefly, total RNA was prepared from the cell culture by Trizol (Thermo fisher Scientific) and quantified by Nano drop ND-1000 (Thermo fisher scientific). The ratio of absorbance at optical density (OD) 260 divided by OD 280 more than 1.8 indicated the adequate purity for further testing. After that, RNA was converted into cDNA by Reverse Transcription System and quantitative polymerase chain reaction (qPCR) was performed using SYBR Green master mix (Applied biosystem, Foster city, CA, USA), cDNA template and target primers based on ΔΔCT method with β-actin as a housekeeping gene. Primers of miR-146a, miR-223 were hsa-miR-146a-5p (Ambion, Cat.4464066, ID.MC10722); mmu-miR-223, ID (Ambion, Cat.4464066, ID.MC14755). In addition, the nucleotide sequences of primers for the analysis of M1 macrophage polarization (*iNOS*, *TNF-a* and *IL-1β*), M2 macrophage polarization (*Arginase-1*, *FIZZ-1* and *TGF-β*) and glycolysis pathway (Integrated DNA Technologies, Coralville, IA, USA) are demonstrated in Table 1.

**Table 1:** List of primers for macrophage polarization and glycolysis pathway are demonstrated.

Primers		
**Macrophage polarization**		
β-actin	Forward	5’-CGGTTCCGATGCCCTGAGGCTCTT-3’
	Reward	5’-CGTCACACTTCATGATGGAATTGA-3’
Inducible nitric oxide synthase (*iNOS*)	Forward	5’-CCCTTCCGAAGTTTCTGGCAGCAGC-3’
	Reward	5’-GGCTGTCAGAGCCTCGTGGCTTTG-3’
Arginase 1 (*Arg-1*)	Forward	5’-CAGAAGAATG GAAGAGTCAG-3’
	Reward	5’-CAGATATGCA GGGA GTCACC-3’
Tumor necrosis factor α (*TNF-α*)	Forward	5’-CCTCACACTCAGATCATCTTCTC-3’
	Reward	5’-AGATCCATGCCG TTGGCCAG-3’
Interleukin-1β (*IL-1β*)	Forward	5’-GAAATGCCACCTTTTGACAGTG-3’
	Reward	5’-TGGATGCTCTCATCAGGACAG-3’
Resistin-like molecule-α (*Fizz-1*)	Forward	5’-GCCAGGTCCTGGAACCTTTC-3’
	Reward	5’-GGAGCAGGGAGATGCAGATGAG-3’
Transforming growth factor-β (*TGF-β*)	Forward	5’-CAGAGCTGCGCTTGCAGAG-3’
	Reward	5’-GTCAGCAGCCGGTTACCAAG-3’
**Glycolysis pathway analysis**		
Solute carrier family 2,facilitated glucose transporter member 10 (*SLC2a10*)	Forward	5’-CAGTTCGGCTATAACACTGGTG-3’
	Reward	5’-GCCCCCGACAGAGAAGATG -3’
Latate dehydrogenase A (*ldhA*)	Forward	5’-CATTGTCAAGTACAGTCCACACT -3’
	Reward	5’-TTCCAATTACTCGGTTTTTGGGA -3’
Phosphofructokisnase (*PFK*)	Forward	5’-GGAGGCGAGAACATCAAGCC -3’
	Reward	5’-CGGCCTTCCCTCGTAGTGA -3’
Pyruvate dehydrogenase kinase-1 (*PDK1*)	Forward	5’-GGACTTCGGGTCAGTGAATGC -3’
	Reward	5’-TCCTGAGAAGATTGTCGGGGA -3’
Hypoxia-inducible factor 1α (*HIF-1*α)	Forward	5’-AGCTTCTGTTATGAGGCTCACC -3’
	Reward	5’-TGACTTGATGTTCATCGTCCTC -3’

### Mitochondrial DNA (mtDNA), total cellular ATP and extracellular flux analysis

Total DNA from cells was extracted by Tissue Genomic DNA extraction mini kit (Favorgen Biotech, Wembley, WA, Australia) as previously published [39]. Briefly, DNA quantification was determined by Nano drop ND-100 (Thermo scientific) and mtDNA copy number was measured by qPCR with cellular DNA template, Mastermix 1xKAPA fast SYBR Green (KAPA Biosystems, Wilmington, MA, USA) and primers of mitochondrial encoded mtDNA (Favorgen Biotech). Quantification of mtDNA was analyzed by ΔΔCT method normalized by the expression of house-keeping gene β2-microglobulin (β2M) with the following sequence, forward, 5’-TTCTGGTGCTTGTCTCACTGA-3′, reverse, 5’-CAGTATGTTCGGCTTCCCATTC-3′. In addition, ATP content in 2x10^4^ cells/ well was determined by the Luminescent ATP Detection Assay (Abcam, Cambridge, UK) according to the manufacturer’s protocol [39]. In addition, energy metabolism profiles with estimation of glycolysis and mitochondrial oxidative phosphorylation with extracellular acidification rate (ECAR) and oxygen consumption rate (OCR) were performed, respectively, by Seahorse XFp Analyzers (Agilent, Santa Clara, CA, USA) upon the miR-transfected RAW264.7 at 1x10^4^ cells/ well by Seahorse Wave 2.6 software as previously described [29]. Glycolysis and mitochondrial parameters was calculated using the generator program report of XF Glycolysis stress test and XF Cell mito stress test, respectively, based on these following equations; Glycolysis = ECAR between glucose and oligomycin–ECAR before glucose administration, glycolysis capacity = ECAR between oligomycin and 2-Deoxy-d-glucose (2-DG)–ECAR before glucose administration, glycolysis reserve = ECAR between oligomycin and 2-DG—ECAR between glucose and oligomycin, basal respiration = OCR before oligomycin–OCR after antimycin A/ rotinone, respiratory capacity (maximal respiration) = OCR between Carbonyl cyanide-4-(trifluoromethoxy)-phenylhydrazone (FCCP) and antimycin A/ rotinone–OCR after antimycin A/ rotinone and respiratory reserve = OCR between FCCP and antimycin A/ rotinone–OCR before oligomycin.

### Statistical analysis

GraphPad Prism 5.0 (GraphPad Software, Inc., San Diego, CA, USA) was used for all statistical analyses. Differences between groups were calculated by Student’s t-test or one-way analysis of variance (ANOVA) followed by Tukey’s analysis for the comparison of two or more experimental groups, respectively. *In vitro* data were based on independent triplicate experiments and represented by mean ± standard error (SE). A p value less than 0.05 was considered as statistically significant.

## Results

The over-expression of miR-223 facilitated M2 macrophage polarization in macrophages (RAW 246.5) through the inhibition of several genes in glycolysis pathway. In addition, the adoptive transfer of miR-223 over-expressed macrophages with IL-4 pre-conditioning attenuated LPS-induced sepsis mouse model.

### The over-expression of miR-223 in macrophages reduced the characteristics of M1 polarization and decreased pro-inflammatory cytokines in cell-supernatant

**Fig 2:**
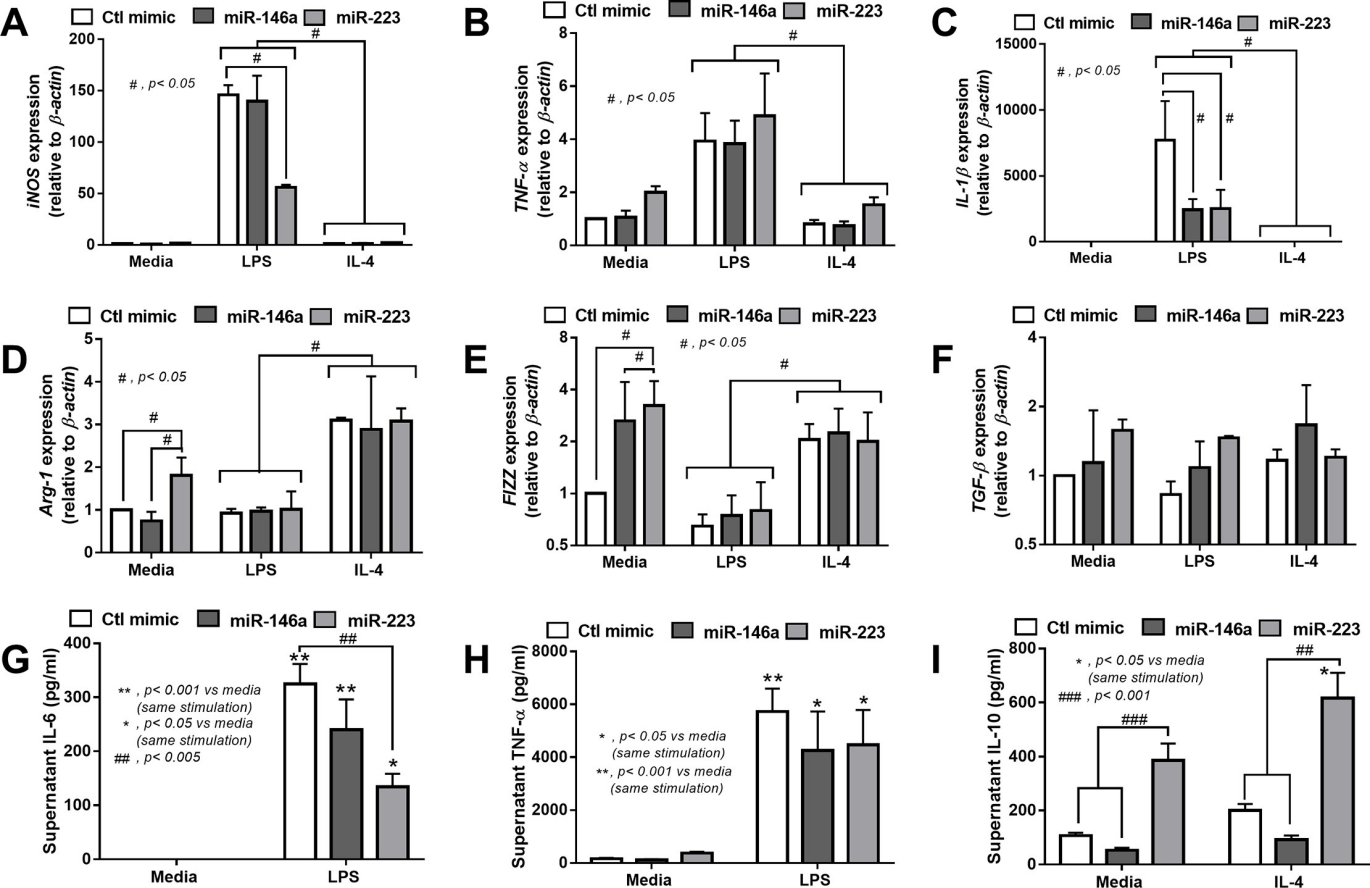
Macrophage anti-inflammatory property after the over-expression of miR-223 is higher than miR-146a. The characteristics of M1 macrophage polarization as determined by the expression of *iNOS*, *TNF-α* and *IL-1β* (A-C), and M2 macrophage polarization as evaluated by the expression of *Arg-1*, *Fizz* and *TGF-β* (D-F), of macrophages with the over-expression of control mimic (Ctl mimic), miR-146a and miR-223 after the stimulation with LPS and IL-4, the stimulator of M1 and M2 macrophage polarization, respectively, are demonstrated. In addition, the supernatant pro-inflammatory cytokines, IL-6 and TNF-α (G, H), after LPS stimulation and an anti-inflammatory cytokine, IL-10 (I), after IL-4 activation, from these macrophages are demonstrated.

**Fig 3:**
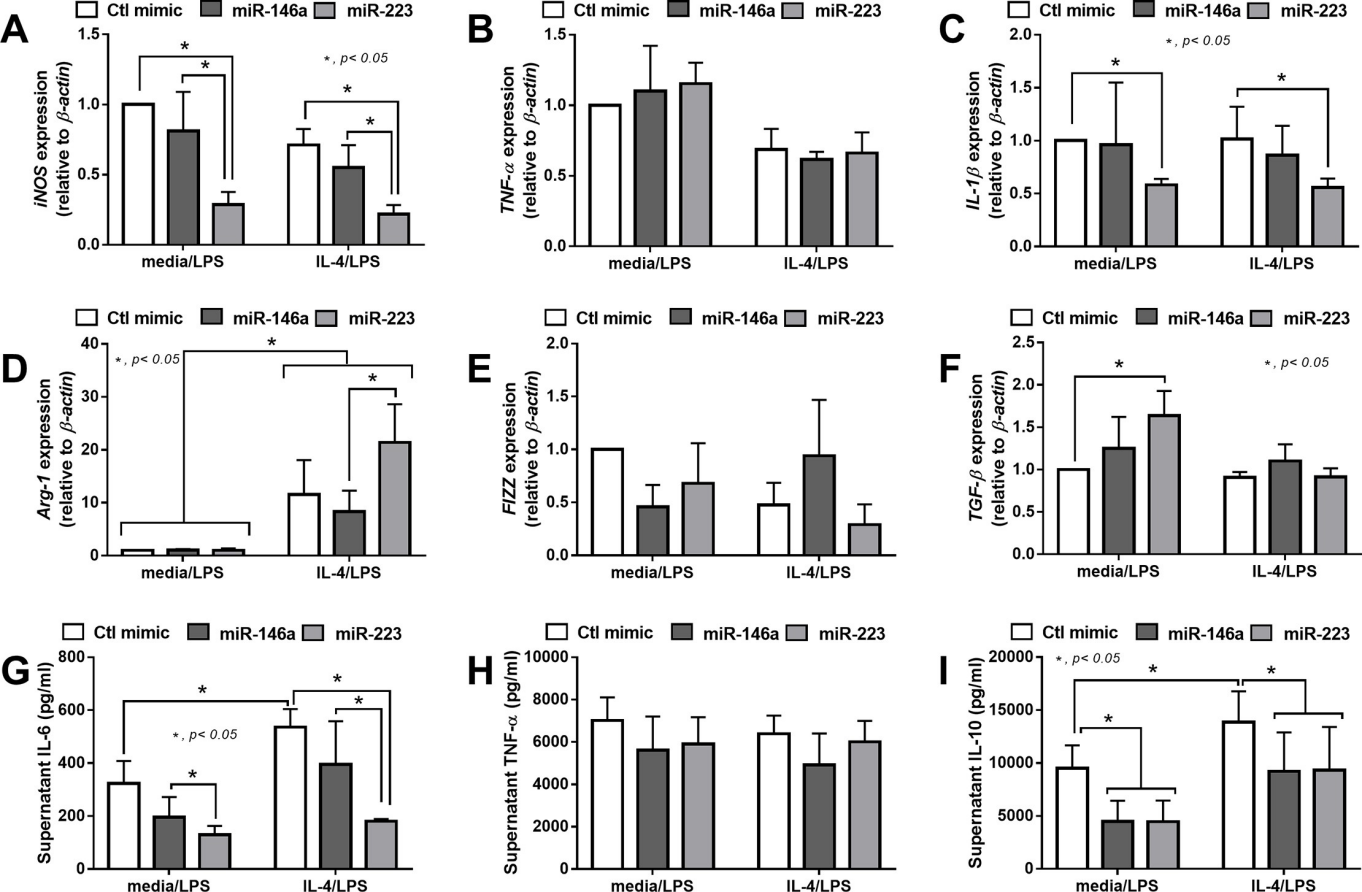
Over-expression of miR-223 maintains IL-4 induced anti-inflammatory characteristics of macrophages after LPS-induction better than miR-146a over-expression. The characteristics of M1 macrophage polarization as determined by the expression of *iNOS*, *TNF-α* and *IL-1β* (A-C), and M2 macrophage polarization as evaluated by the expression of *Arg-1*, *Fizz* and *TGF-β* (D-F), of macrophages with the over-expression of control mimic (Ctl mimic), miR-146a and miR-223 that stimulated with IL-4, the M2 macrophage polarization stimulator, prior to the stimulation with LPS, M1 macrophage-polarization stimulator, (IL-4/LPS) or control media prior to LPS (media/LPS) are demonstrated. In addition, the supernatant cytokines, IL-6, TNF-α and IL-10 (G-I), of these macrophages are demonstrated.

Because of the known anti-inflammatory effect of both miR-223 and miR-146a in macrophages [27], both miRs were transfected in RAW264.7 before the induction of M1 and M2 polarization by LPS and IL-4, respectively. As such, the overexpression of miR-223 down-regulated the gene expression of M1 biomarkers including *iNOS* and *IL-1β*, but not *TNF-α*, while the over-expressed miR-146a down-regulated only *IL-1β* (Fig 2A–2C). In parallel, the overexpression of miR-223 induced M2 polarization as demonstrated by the up-regulation of *Arg-1* and *Fizz*, but not *TGF-β*, and the over-expressed miR-146a up-regulated only *TGF-β* (Fig 2D–2F). However, the expression of both miRs did not further enhance the characteristics of IL-4 activated M2 polarization, as determined by the expression of several biomarkers, in comparison with IL-4 induced control mimic cells (Fig 2D–2F). In addition, the anti-inflammatory property of miR-223 was demonstrated by i) the reduction of supernatant IL-6, but not TNF-α, after LPS activation (Fig 2G and 2H) and ii) the induction of supernatant IL-10 after IL-4 stimulation (Fig 2I). On the other hand, the over-expression of miR-146a did not demonstrate an anti-inflammatory effect as evaluated by supernatant cytokines (Fig 2G–2I). Because the conversion from IL-4 activated M2 polarization into pro-inflammatory macrophages by LPS was previously mentioned [40, 41], the property to maintain M2 polarization after IL-4 stimulation in miR-223 overexpressed macrophages was tested. Accordingly, while IL-4 stimulation alone induced very low expression of *iNOS*, *TNF-α* and *IL-1β* compared with LPS stimulation (Fig 2A–2C), LPS activation in IL-4 pre-conditioning macrophages (IL-4/ LPS) demonstrated a similar level of these genes in comparison with LPS stimulation alone (media/ LPS) (Fig 3A–3C). However, miR-223 over-expressed macrophages with IL-4 pre-conditioning (IL-4/ LPS) enhanced only some pro-inflammatory characteristics but still demonstrated lower *iNOS* expression, higher *Arg-1* expression and lower supernatant IL-6 (Fig 3) compared with the miR-146a over-expressed cells.

### The inhibition of enzyme in glycolysis pathway by the miR-223 over-expression induced resistance against LPS-enhanced M1 polarization

**Fig 4:**
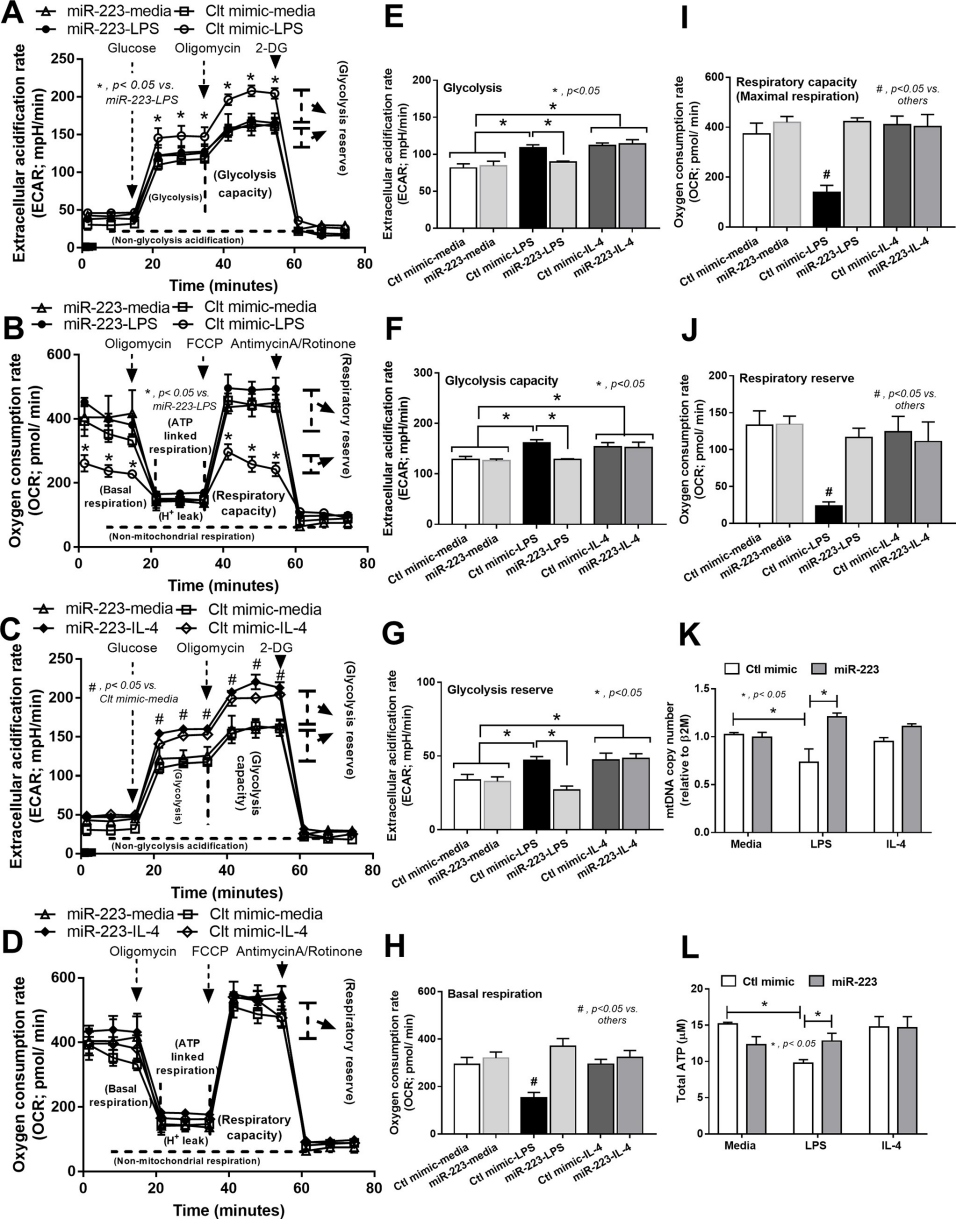
Over-expression of miR-223 in LPS-activated macrophages reduces glycolysis activity and improves mitochondrial function as determined by extracellular flux analysis. The extracellular flux analysis pattern of macrophages over-expressed by miR-223 or control mimic (Ctl-mimic) as evaluated by extracellular acidification rate of glucose stress test for glycolysis pathway analysis and oxygen consumption rate of mitochondrial stress test for mitochondrial pathway analysis after the stimulation by LPS versus control media (A, B) or IL-4 versus control media (C, D) with the comparison in column bar graph of glucose stress test parameters (glycolysis, respiratory capacity and glycolysis reserve) (E-G) and mitochondrial stress test parameters (basal respiration, respiratory capacity and respiratory reserve) (H-J) are demonstrated. In addition, the mitochondrial content as analyzed by the copy number of mitochondria (mtDNA) (K) and the ATP production (L) of macrophages over-expressed with Ctl-mimic or miR-223 after the stimulation with LPS, IL-4 or control media are demonstrated. Of note, the extracellular flux analysis pattern of miR-223-media is not showed due to the similarity to Ctl-mimic-media.

Because the alteration of immunometabolism is, at least in part, responsible for the expression of pro- or anti- inflammatory characteristics of macrophages [42], the metabolism profiles are explored by the extracellular flux analysis. As such, LPS induced the glycolysis pathway as indicated by increased glycolysis area, glycolysis capacity and glycolysis reserve (Fig 4A; Ctl mimic-LPS vs. Ctl mimic-media; 4E–4G). In parallel, LPS reduced the mitochondrial pathway as indicated by reduced basal respiration, respiratory capacity and respiratory reserve (Fig 4B; Ctl mimic-LPS vs. Ctl mimic-media, 4H–4J). However, the patterns of glycolysis and mitochondrial responses in miR-223 over-expressed macrophages after LPS stimulation was similar to control (Fig 4A and 4B; Ctl mimic-media vs. miR-223-LPS, 4E–4J) implying the resistance of miR-223 over-expressed cells against the direction of LPS stimulation. On the other hand, IL-4 (Ctl mimic IL-4) also increased glycolysis activity compared with control (Ctl mimic-media) (Fig 4C and 4E–4G) without the alteration in mitochondrial activity (Fig 4D and 4H–4J). Moreover, LPS also reduced mitochondrial number, as determined by mitochondrial DNA analysis, and decreased total ATP while IL-4 did not change these parameters (Fig 4K and 4L). Interestingly, mitochondrial DNA and ATP production in miR-223 over-expressed cells with LPS stimulation did not differ from the control macrophages (Fig 4K and 4L). Of note, the profile of extra-cellular flux analysis in miR-223 over-expressed macrophages without LPS (miR-223-media) did not differ from the control mimic (Ctl mimic-media). These data supported that LPS induced glycolysis, suppressed mitochondrial function and reduced ATP production in macrophages but were attenuated by miR-223 over-expression.

**Fig 5:**
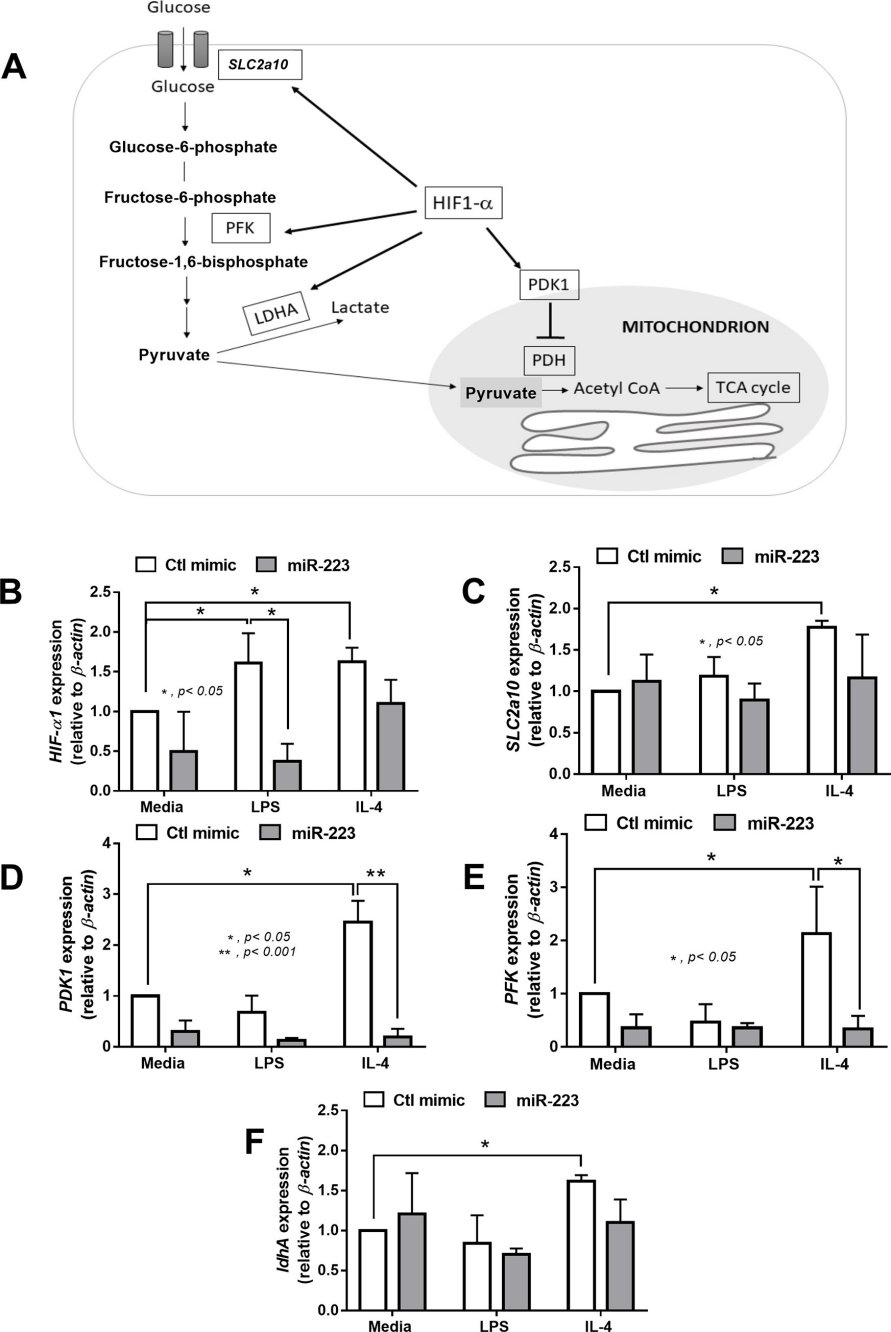
Over-expression of miR-223 in LPS-activated macrophages reduces *HIF-1α* expression, while miR-223 over-expression in IL-4-activated macrophages decreases expression of *PDK-1* and *PFK*. The diagram demonstrates enzymes that associated with glycolysis pathway (A) and the gene expression of these enzymes in macrophages with the over-expression of miR-223 or control mimic (Ctl mimic) after the stimulation with LPS or IL-4 (B-F) are demonstrated. HIF-1α, hypoxia-inducible factor-1α; *SLC2a10*, solute carrier family 2,facilitated glucose transporter member 10; PFK, phosphofructokinase; LDHA, lactate dehydrogenase A; PDK1, pyruvate dehydrogenase kinase 1, PDH: pyruvate dehydrogenase; Acetyl-CoA, Acetyl Coenzyme A; TCA cycle, tri-carboxylic acid cycle or Krebs cycle.

Due to the inhibitory property of miR against the mRNA functions [12], we hypothesize that miR-223 might inhibit some mRNAs in glycolysis pathway. Accordingly, hypoxic inducible factor-1α (HIF-1α) is an enzyme responsible to enhance glycolysis pathway through several mediators including Solute carrier family 2, facilitated glucose transporter member 10 (SLC2a10), phosphofructokinase (PFK), Lactate dehydrogenase A (ldhA) [43] (Fig 5A). Furthermore, HIF-1α also reduces the mitochondrial function through the expression of pyruvate dehydrogenase kinase (PDK1) [44]. Of note, increased PDK1 inhibited pyruvate dehydrogenase (PHD), an enzyme that converts pyruvate into acetyl CoA supplying for Krebs cycle, leads to the reduction of mitochondrial function [44]. As such, the exploration of glycolysis-related genes in macrophages demonstrated that LPS induced only *HIF-1α* expression (Fig 5B), while IL-4 enhanced the expression of several genes including; *HIF-1α*, *SLC2a10*, *PDK1*, *PKF* and *ldhA* (Fig 5C–5F). Although miR-223 over-expression reduced only *HIF-1α* in LPS-activated macrophages (Fig 5A), the LPS-induced glycolysis was inhibited (Fig 4A), implying the major role of *HIF-1α* in the glycolysis pathway. In contrast, miR-223 inhibited both *PDK-1* and *PFK* in IL-4 stimulated-cells (Fig 5E and 5F) without any impact upon extracellular flux pattern (Fig 4C and 4D), suggesting less importance of these enzymes in glycolysis pathway.

### The administration of IL-4 preconditioning macrophages with miR-233 over-expression attenuated sepsis in LPS injection mice

**Fig 6:**
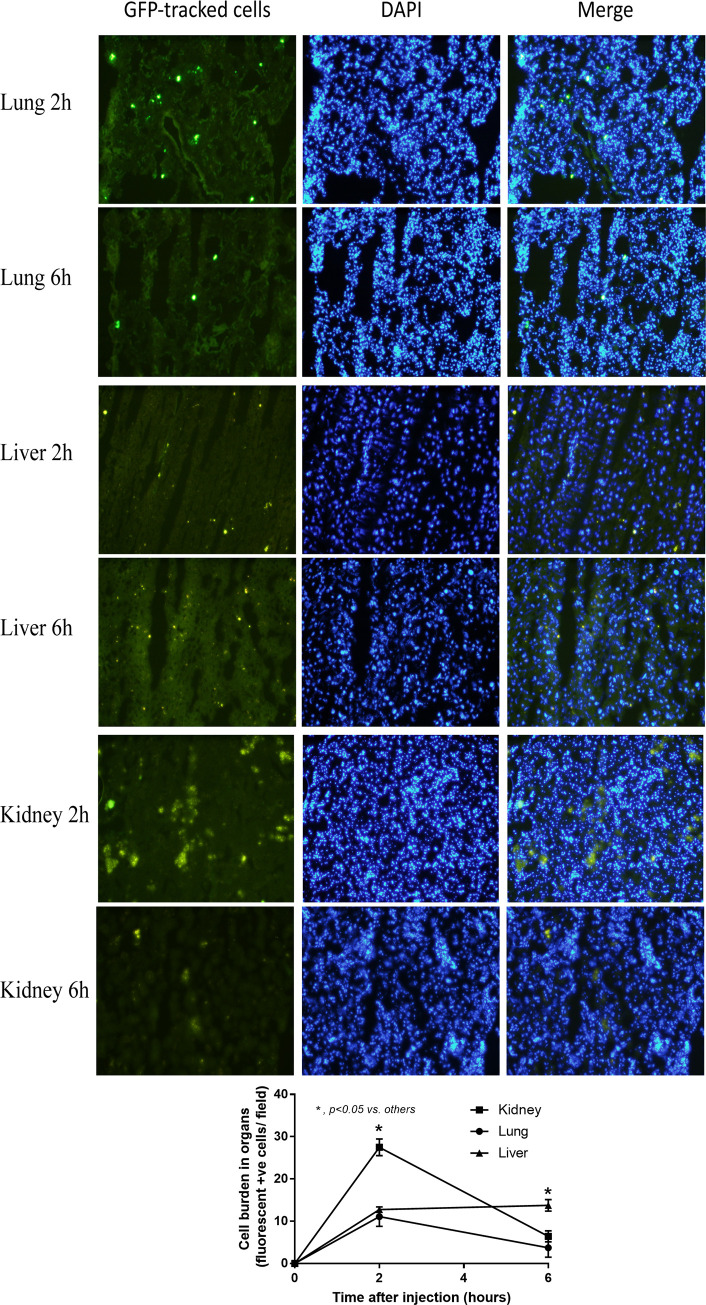
Macrophages are demonstrated in kidney and liver at 2 h and 6 h after administration. The representative pictures of the kinetics of tagged green fluorescent protein (GFP) macrophages after intravenous injection in different organs at different time-points and the analysis of cells burdens in organs are demonstrated. DAPI, 4′, 6-diamidino-2-phenylindole (a DNA fluorescence staining color).

**Fig 7:**
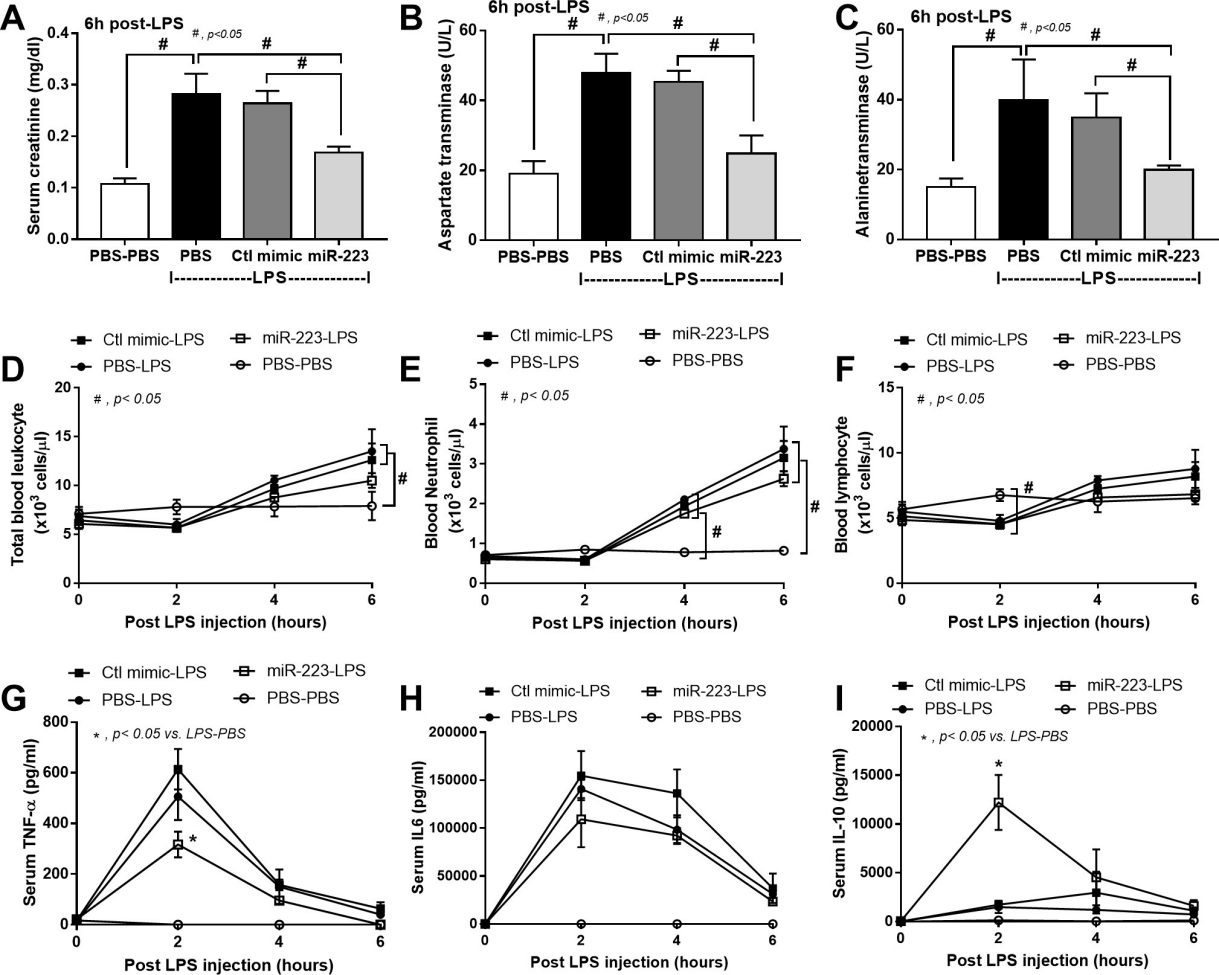
Administration of miR-223 over-expressed macrophages attenuates sepsis in LPS injection mouse model. The characteristics of LPS injection mice after the administration with IL-4-activated-macrophages without gene over-expression (PBS-LPS) or with control mimic over-expression (Ctl mimic-LPS) or with miR-223 over-expression (miR-223-LPS) as evaluated by serum creatinine (A), aspartate transaminase (B), alanine transaminase (C), blood leukocyte evaluation (D-F) and serum cytokines (G-I) are demonstrated (n = 5-7/ group). Of note, only the control mice injected with phosphate buffer solution (PBS) group without LPS stimulation (PBS-PBS) is presented, but not Ctl mimic-PBS and miR-223-PBS, due to the similar values among these 3 control groups.

**Fig 8:**
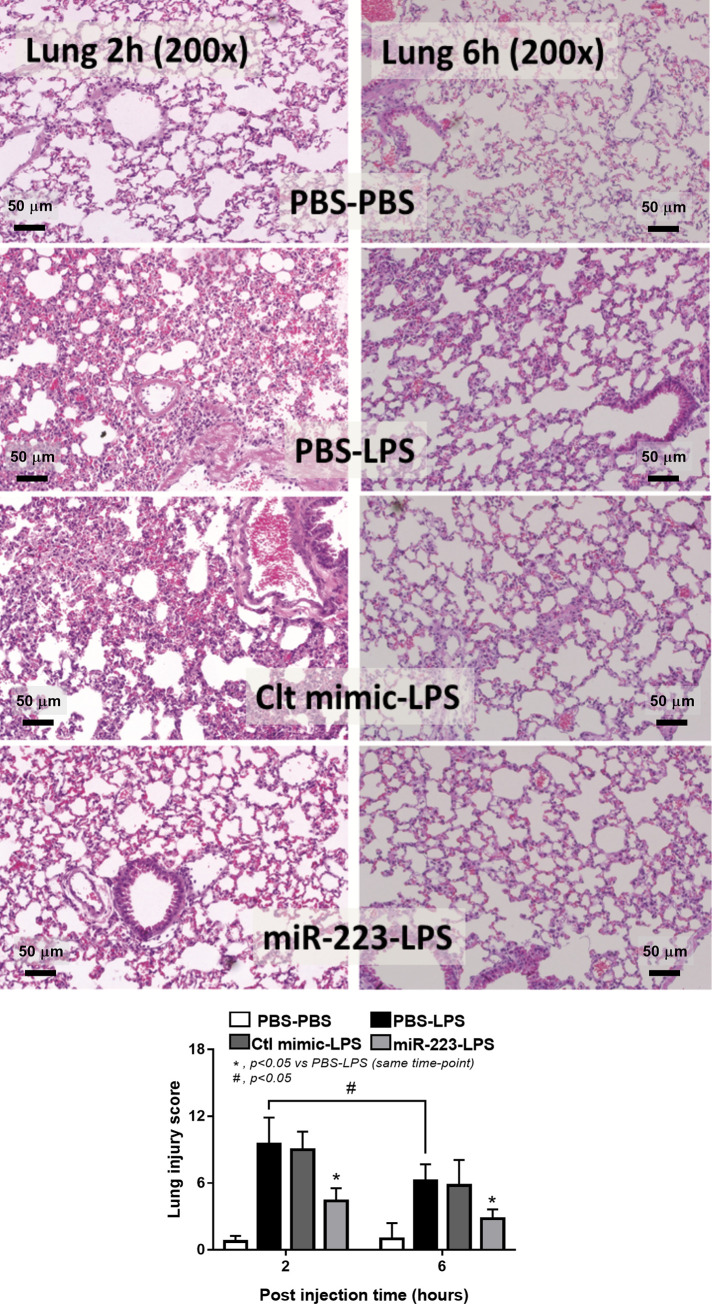
Administration of miR-223 over-expressed macrophages attenuates sepsis-induced lung injury in LPS injection mouse model. The representative of lung histology (A) of LPS administered mice preconditioning with IL-4-activated-macrophages without gene over-expression (PBS-LPS) or with control mimic over-expression (Ctl mimic-LPS) or with miR-223 over-expression (miR-223-LPS) by the semi-quantitative histology score (B) are demonstrated (n = 5-7/ group). Of note, only the control mice injected with phosphate buffer solution (PBS) group without LPS stimulation (PBS-PBS) is presented, but not Ctl mimic-PBS and miR-223-PBS, due to the similar pictures among these 3 control groups.

Because preconditioning with IL-4 in miR-223 over-expressed macrophages demonstrated anti-inflammatory state (Fig 2) that resisted the LPS induced pro-inflammation (Fig 3) possibly through the interference of HIF-1α (Figs 4 and 5), these cells were used *in vivo* following previous publications [31–34]. As such, the intravenous cells administration demonstrated the highest cell accumulation in kidney and in liver at 1 h and 6 h post-injection, respectively (Fig 6). The administration of IL-4 pre-conditioning macrophages with miR-223 over-expression, but not IL-4 stimulated control cells, attenuated the severity of LPS injection model as determined by renal injury (serum creatinine), liver injury (aspartate transaminase and alanine transaminase) (Fig 7A–7C) without therapeutic effect on leukocyte count (Fig 7D–7F). In addition, treatment with miR-223-manipulated cells also reduced pro-inflammatory cytokine (TNF-α), but not IL-6, increased anti-inflammatory cytokine (IL-10) (Fig 7G–7I) and improved lung pathology (Fig 8).

## Discussion

The over-expression of miR-223 in macrophages induced anti-inflammatory responses against LPS stimulation possibly due to the glycolysis interference through reduced *HIF-1α*. The administration of IL-4 stimulated miR-223 over-expressed macrophages attenuated the severity of LPS injection sepsis mouse model.

### Anti-inflammatory property of miR-223 over-expressed macrophages

The hyper-inflammatory responses induced sepsis mortality and the attenuation by anti-inflammatory macrophages are well-known [9, 10]. Due to the multiple inhibitory targets, either complementary or non-complementary sequence mRNA, is possible from a single miR [13], miR is an interesting method to induce anti-inflammatory macrophages. Indeed, both miR-223 and miR-146a (without IL-4) are associated with M2 macrophages polarization [45, 46] through, at least in part, targeting PPARγ [15, 47]. With LPS stimulation, the anti-inflammatory property of miR-223 over-expressed macrophages was more potent than miR-146a over-expressed cells as determined by the reduced expression of *IL-1β* and *iNOS* with decreased supernatant IL-6 and increased supernatant IL-10. Different effects of these miRs suggest the inhibition in different target-molecules. However, previous studies report that IL-4-primed macrophages are easily converted back into the pro-inflammatory macrophages after the subsequent LPS challenge due to the macrophage plasticity [40, 41]. Here, miR-223 over-expression prevented the conversion back into the pro-inflammatory state with the subsequent LPS stimulation but miR-223 did not enhance the expression of IL-4 induced anti-inflammatory genes.

### The interference of glycolysis pathway by miR-223 induced anti-inflammatory macrophages

The signatures of immunometabolic pathway of M1 and M2 polarized macrophages are glycolysis and mitochondrial oxidative phosphorylation, respectively [21]. As such, the induction of macrophage polarization by immunometabolic alteration using miRs is mentioned [22]. Here, LPS down regulated mitochondrial oxidative phosphorylation, along with the reduction of mtDNA and ATP production, with glycolysis enhancement while IL-4 stimulated only the glycolysis pathway supporting previous publications [48–50]. While glycolysis is not a major metabolic pathway in M2 macrophages polarization [51], glycolysis is required for the regular M2 macrophage functions [48] and the interference on glycolysis inhibits M2 macrophage polarization [49]. Because i) LPS increased only *HIF-1α* while IL-4 enhanced *HIF-1α* with other genes and ii) miR-223 over-expression, that mainly inhibited *HIF-1α*, turned the immunometabolic characteristics of LPS-stimulated cells into the neutral state but no effect on IL-4-stimulated cells. These data implied a possible key role of *HIF-1α* in glycolysis pathway after LPS activation [52]. Indeed, the impact of *HIF-1α* upon glycolysis and M1 polarization is previously mentioned including; i) *HIF-1α* expression up-regulates several enzymes in glycolysis [43], ii) *HIF-1α* overexpression enhances glycolysis and increases inflammatory cytokine production in macrophages [53] and iii) *HIF-1α* deficient macrophages did not produce cytokines after LPS stimulation [54]. Thus the interference of *HIF-1α* expression might be one of the important mechanisms of miR-223-induced anti-inflammatory macrophages.

In addition, from the previous reports, miR-223 also induced anti-inflammatory properties through the interference of other mechanisms such as STAT3, Nfat5 and PPARγ [15, 26] implying the multiple targets of miR-223 for anti-inflammatory induction. On the other hand, miR-223 over-expression reduced the expression of *PFK* and *PDK-1*, but did not alter immunometabolic characteristics, of IL-4 stimulated cells implying that these enzymes are not the main enzymes used for cell-energy generation during IL-4 induced M2 macrophage polarization.

Of note, miR-223 regulation on *HIF-1α* translation is nicely mentioned though the analysis of 3-prime un-translated region (3’ UTR) [24, 25]. In addition, LPS and IL-4 possibly mediate *HIF-1α* expression differently. Our working hypothesis is that while LPS enhances *HIF-1α* through LPS-induced reactive oxygen species [55], IL-4 activates *HIF-1α* via Akt-mTORC [48, 49], an upstream signaling of *HIF-1α* [56]. As such, LPS and IL-4 enhanced glycolysis as demonstrated by extracellular flux analysis and genes of glycolysis pathway mainly through *HIF-1α* and *HIF-1α* with other several genes, respectively (Fig 4). It seems that miR-223 targets only on *HIF-1α* in LPS-enhanced glycolysis, but binds to *HIF-1α* together with other several genes in IL-4-enhanced glycolysis. Indeed, the predicted-targets of miR-223 from the database of miRNAWalk 2.0 and TargetScan.org version 7.2, the comprehensive database on the predicted and validated targets of human and murine miR [57], was including both *HIF-1α* and *PFK*. Here, we demonstrated that LPS increased *HIF-1α* while IL-4 induced *HIF-1α*, *PDK-1* and *PFK*. Hence, miR-223 blocked only *HIF-1α* in LPS stimulation but targeted on *HIF-1α*, *PDK-1* and *PFK* in IL-4 induction, resulted in the higher inhibitory potency on LPS-derived *HIF-1α* compared with IL-4 induced *HIF-1α*. More studies in this topic are interesting.

### The administration of IL-4 stimulated miR-223 over-expressed macrophages, a proof of concept for anti-inflammatory treatment in sepsis

Because of the potent anti-inflammatory property and the resistance against LPS induced macrophage-conversion of miR-223 over-expression, IL-4 stimulated miR-223 over-expressed macrophages were tested in LPS injection mouse model as a proof of concept for sepsis treatment. The injected cells distributed in lung, liver and kidney for at least 6 h after the administration. Immune-mediated rejection of C57BL/6 mice toward the administered RAW264.7 cell, a cell line from BALB/c mice, might be responsible for the cell depletion which could be improved in the clinical translation. Nevertheless, the treatment attenuated the injury in several organs as evaluated by lung histology, liver enzymes and serum creatinine with decreased systemic inflammation as demonstrated by decreased TNF-α and increased IL-10 in serum at 6 h post LPS administration, a time-point with the highest injury of the model. Interestingly, administration of IL-4 stimulated macrophages without miR-223 over-expression did not demonstrate any beneficial effects in LPS injection model, perhaps due to the conversion back into pro-inflammatory state of the injected-cells by LPS stimulation as previously mentioned [40, 41, 58]. In addition, the higher pro-inflammatory responses by LPS stimulation in IL-4 pre-conditioning macrophages in comparison with LPS-stimulated control macrophages is demonstrated here (Fig 4G) supporting several previous publications [40, 59, 60], possibly through IL-4 activated STAT-6 [41], which is a possible limitation of cell therapy in sepsis. Hence, the plasticity of macrophages in cell-based therapy in sepsis is an important issue for further studies. In translational aspect, the injected cells that distribute in several organs might be responsible for direct local inflammatory control within the organs and is possibly an interesting adjunctive therapy in sepsis. Cell-based therapy has been currently developed for cancer treatment [61, 62] and some of these therapies might also be appropriated for use in sepsis. More studies are interesting.

In conclusion, miR-223 over-expression induced anti-inflammation through the down-regulation of *HIF-1α* that inhibited the glycolysis pathway. The anti-inflammatory induction by IL-4 in miR-223 over-expressed macrophages prevented the back-conversion into pro-inflammatory macrophage after LPS stimulation. These cells are proposed as a candidate for cell therapy during the pro-inflammatory phase of sepsis.

## References

[1] Singer M, Deutschman CS, Seymour CW, Shankar-Hari M, Annane D, Bauer M, et al The Third International Consensus Definitions for Sepsis and Septic Shock (Sepsis-3). JAMA. 2016;315(8):801–10. 10.1001/jama.2016.0287 26903338 PMC4968574

[2] Doi K, Leelahavanichkul A, Yuen PS, Star RA. Animal models of sepsis and sepsis-induced kidney injury. J Clin Invest. 2009;119(10):2868–78. 10.1172/JCI39421 19805915 PMC2752080

[3] Rosen DA, Seki SM, Fernandez-Castaneda A, Beiter RM, Eccles JD, Woodfolk JA, et al Modulation of the sigma-1 receptor-IRE1 pathway is beneficial in preclinical models of inflammation and sepsis. Sci Transl Med. 2019;11(478). 10.1126/scitranslmed.aau5266 PMC6936250 30728287

[4] Riedemann NC, Ward PA. Anti-inflammatory strategies for the treatment of sepsis. Expert Opin Biol Ther. 2003;3(2):339–50. 10.1517/14712598.3.2.339 12662146

[5] Hotchkiss RS, Moldawer LL, Opal SM, Reinhart K, Turnbull IR, Vincent JL. Sepsis and septic shock. Nat Rev Dis Primers. 2016;2:16045 10.1038/nrdp.2016.45 28117397 PMC5538252

[6] Varol C, Mildner A, Jung S. Macrophages: development and tissue specialization. Annu Rev Immunol. 2015;33:643–75. 10.1146/annurev-immunol-032414-112220 25861979

[7] Murray PJ. Macrophage Polarization. Annu Rev Physiol. 2017;79:541–66. 10.1146/annurev-physiol-022516-034339 27813830

[8] Hamidzadeh K, Christensen SM, Dalby E, Chandrasekaran P, Mosser DM. Macrophages and the Recovery from Acute and Chronic Inflammation. Annu Rev Physiol. 2017;79:567–92. 10.1146/annurev-physiol-022516-034348 27959619 PMC5912892

[9] Taratummarat S, Sangphech N, Vu CTB, Palaga T, Ondee T, Surawut S, et al Gold nanoparticles attenuates bacterial sepsis in cecal ligation and puncture mouse model through the induction of M2 macrophage polarization. BMC Microbiol. 2018;18(1):85 10.1186/s12866-018-1227-3 30119646 PMC6098657

[10] Nemeth K, Leelahavanichkul A, Yuen PS, Mayer B, Parmelee A, Doi K, et al Bone marrow stromal cells attenuate sepsis via prostaglandin E(2)-dependent reprogramming of host macrophages to increase their interleukin-10 production. Nat Med. 2009;15(1):42–9. 10.1038/nm.1905 19098906 PMC2706487

[11] Essandoh K, Li Y, Huo J, Fan GC. MiRNA-Mediated Macrophage Polarization and its Potential Role in the Regulation of Inflammatory Response. Shock. 2016;46(2):122–31. 10.1097/SHK.0000000000000604 26954942 PMC4949115

[12] Gebert LFR, MacRae IJ. Regulation of microRNA function in animals. Nat Rev Mol Cell Biol. 2019;20(1):21–37. 10.1038/s41580-018-0045-7 30108335 PMC6546304

[13] Selbach M, Schwanhausser B, Thierfelder N, Fang Z, Khanin R, Rajewsky N. Widespread changes in protein synthesis induced by microRNAs. Nature. 2008;455(7209):58–63. 10.1038/nature07228 18668040

[14] Wu XQ, Dai Y, Yang Y, Huang C, Meng XM, Wu BM, et al Emerging role of microRNAs in regulating macrophage activation and polarization in immune response and inflammation. Immunology. 2016;148(3):237–48. 10.1111/imm.12608 27005899 PMC4913289

[15] Ying W, Tseng A, Chang RC, Morin A, Brehm T, Triff K, et al MicroRNA-223 is a crucial mediator of PPARgamma-regulated alternative macrophage activation. J Clin Invest. 2015;125(11):4149–59. 10.1172/JCI81656 26436647 PMC4639972

[16] Wang JF, Yu ML, Yu G, Bian JJ, Deng XM, Wan XJ, et al Serum miR-146a and miR-223 as potential new biomarkers for sepsis. Biochem Biophys Res Commun. 2010;394(1):184–8. 10.1016/j.bbrc.2010.02.145 20188071

[17] Taganov KD, Boldin MP, Chang KJ, Baltimore D. NF-kappaB-dependent induction of microRNA miR-146, an inhibitor targeted to signaling proteins of innate immune responses. Proc Natl Acad Sci U S A. 2006;103(33):12481–6. 10.1073/pnas.0605298103 16885212 PMC1567904

[18] Gao M, Wang X, Zhang X, Ha T, Ma H, Liu L, et al Attenuation of Cardiac Dysfunction in Polymicrobial Sepsis by MicroRNA-146a Is Mediated via Targeting of IRAK1 and TRAF6 Expression. J Immunol. 2015;195(2):672–82. 10.4049/jimmunol.1403155 26048146 PMC4490963

[19] O'Neill LA, Kishton RJ, Rathmell J. A guide to immunometabolism for immunologists. Nat Rev Immunol. 2016;16(9):553–65. 10.1038/nri.2016.70 27396447 PMC5001910

[20] Domblides C, Lartigue L, Faustin B. Metabolic Stress in the Immune Function of T Cells, Macrophages and Dendritic Cells. Cells. 2018;7(7). 10.3390/cells7070068 PMC6070887 29966302

[21] Thapa B, Lee K. Metabolic influence on macrophage polarization and pathogenesis. BMB Rep. 2019;52(6):360–72. 10.5483/BMBRep.2019.52.6.140 31186085 PMC6605523

[22] Ouimet M, Ediriweera HN, Gundra UM, Sheedy FJ, Ramkhelawon B, Hutchison SB, et al MicroRNA-33-dependent regulation of macrophage metabolism directs immune cell polarization in atherosclerosis. J Clin Invest. 2015;125(12):4334–48. 10.1172/JCI81676 26517695 PMC4665799

[23] Subramaniam S, Jeet V, Clements JA, Gunter JH, Batra J. Emergence of MicroRNAs as Key Players in Cancer Cell Metabolism. Clin Chem. 2019;65(9):1090–101. 10.1373/clinchem.2018.299651 31101638

[24] Yang L, Li Y, Wang X, Mu X, Qin D, Huang W, et al Overexpression of miR-223 Tips the Balance of Pro- and Anti-hypertrophic Signaling Cascades toward Physiologic Cardiac Hypertrophy. J Biol Chem. 2016;291(30):15700–13. 10.1074/jbc.M116.715805 27226563 PMC4957053

[25] Zhao Z, Jinde S, Koike S, Tada M, Satomura Y, Yoshikawa A, et al Altered expression of microRNA-223 in the plasma of patients with first-episode schizophrenia and its possible relation to neuronal migration-related genes. Transl Psychiatry. 2019;9(1):289 10.1038/s41398-019-0609-0 31712567 PMC6848172

[26] Chen Q, Wang H, Liu Y, Song Y, Lai L, Han Q, et al Inducible microRNA-223 down-regulation promotes TLR-triggered IL-6 and IL-1beta production in macrophages by targeting STAT3. PLoS One. 2012;7(8):e42971 10.1371/journal.pone.0042971 22937006 PMC3427313

[27] Vergadi E, Vaporidi K, Theodorakis EE, Doxaki C, Lagoudaki E, Ieronymaki E, et al Akt2 deficiency protects from acute lung injury via alternative macrophage activation and miR-146a induction in mice. J Immunol. 2014;192(1):394–406. 10.4049/jimmunol.1300959 24277697

[28] Leelahavanichkul A, Worasilchai N, Wannalerdsakun S, Jutivorakool K, Somparn P, Issara-Amphorn J, et al Gastrointestinal Leakage Detected by Serum (1—>3)-beta-D-Glucan in Mouse Models and a Pilot Study in Patients with Sepsis. Shock. 2016;46(5):506–18. 10.1097/SHK.0000000000000645 27172153

[29] Ondee T, Gillen J, Visitchanakun P, Somparn P, Issara-Amphorn J, Dang Phi C, et al Lipocalin-2 (Lcn-2) Attenuates Polymicrobial Sepsis with LPS Preconditioning (LPS Tolerance) in FcGRIIb Deficient Lupus Mice. Cells. 2019;8(9). 10.3390/cells8091064 PMC6769833 31514375

[30] Ondee T, Jaroonwitchawan T, Pisitkun T, Gillen J, Nita-Lazar A, Leelahavanichkul A, et al Decreased Protein Kinase C-beta Type II Associated with the Prominent Endotoxin Exhaustion in the Macrophage of FcGRIIb-/- Lupus Prone Mice is Revealed by Phosphoproteomic Analysis. Int J Mol Sci. 2019;20(6). 10.3390/ijms20061354 PMC6472018 30889825

[31] Kong XN, Yan HX, Chen L, Dong LW, Yang W, Liu Q, et al LPS-induced down-regulation of signal regulatory protein {alpha} contributes to innate immune activation in macrophages. J Exp Med. 2007;204(11):2719–31. 10.1084/jem.20062611 17954568 PMC2118489

[32] Vinuesa E, Hotter G, Jung M, Herrero-Fresneda I, Torras J, Sola A. Macrophage involvement in the kidney repair phase after ischaemia/reperfusion injury. J Pathol. 2008;214(1):104–13. 10.1002/path.2259 17973244

[33] Perske C, Lahat N, Sheffy Levin S, Bitterman H, Hemmerlein B, Rahat MA. Loss of inducible nitric oxide synthase expression in the mouse renal cell carcinoma cell line RENCA is mediated by microRNA miR-146a. Am J Pathol. 2010;177(4):2046–54. 10.2353/ajpath.2010.091111 20709800 PMC2947298

[34] Gerasimovskaya E, Kratzer A, Sidiakova A, Salys J, Zamora M, Taraseviciene-Stewart L. Interplay of macrophages and T cells in the lung vasculature. Am J Physiol Lung Cell Mol Physiol. 2012;302(10):L1014–22. 10.1152/ajplung.00357.2011 22387295 PMC3362259

[35] Leelahavanichkul A, Somparn P, Bootprapan T, Tu H, Tangtanatakul P, Nuengjumnong R, et al High-dose ascorbate with low-dose amphotericin B attenuates severity of disease in a model of the reappearance of candidemia during sepsis in the mouse. Am J Physiol Regul Integr Comp Physiol. 2015;309(3):R223–34. 10.1152/ajpregu.00238.2014 25994956 PMC4525325

[36] Gupta N, Su X, Popov B, Lee JW, Serikov V, Matthay MA. Intrapulmonary delivery of bone marrow-derived mesenchymal stem cells improves survival and attenuates endotoxin-induced acute lung injury in mice. J Immunol. 2007;179(3):1855–63. 10.4049/jimmunol.179.3.1855 17641052

[37] Li J, Li D, Liu X, Tang S, Wei F. Human umbilical cord mesenchymal stem cells reduce systemic inflammation and attenuate LPS-induced acute lung injury in rats. J Inflamm (Lond). 2012;9(1):33. 22974286 10.1186/1476-9255-9-33 PMC3502090

[38] Tangtanatakul P, Thammasate B, Jacquet A, Reantragoon R, Pisitkun T, Avihingsanon Y, et al Transcriptomic profiling in human mesangial cells using patient-derived lupus autoantibodies identified miR-10a as a potential regulator of IL8. Sci Rep. 2017;7(1):14517 10.1038/s41598-017-15160-8 29109423 PMC5673966

[39] Thim-Uam A, Surawut S, Issara-Amphorn J, Jaroonwitchawan T, Hiengrach P, Chatthanathon P, et al Leaky-gut enhanced lupus progression in the Fc gamma receptor-IIb deficient and pristane-induced mouse models of lupus. Sci Rep. 2020;10(1):777 10.1038/s41598-019-57275-0 31964918 PMC6972921

[40] Rajaiah R, Perkins DJ, Polumuri SK, Zhao A, Keegan AD, Vogel SN. Dissociation of endotoxin tolerance and differentiation of alternatively activated macrophages. J Immunol. 2013;190(9):4763–72. 10.4049/jimmunol.1202407 23543762 PMC3633613

[41] Major J, Fletcher JE, Hamilton TA. IL-4 pretreatment selectively enhances cytokine and chemokine production in lipopolysaccharide-stimulated mouse peritoneal macrophages. J Immunol. 2002;168(5):2456–63. 10.4049/jimmunol.168.5.2456 11859138

[42] Koelwyn GJ, Corr EM, Erbay E, Moore KJ. Regulation of macrophage immunometabolism in atherosclerosis. Nat Immunol. 2018;19(6):526–37. 10.1038/s41590-018-0113-3 29777212 PMC6314674

[43] Li XB, Gu JD, Zhou QH. Review of aerobic glycolysis and its key enzymes—new targets for lung cancer therapy. Thorac Cancer. 2015;6(1):17–24. 10.1111/1759-7714.12148 26273330 PMC4448463

[44] Tan Z, Xie N, Cui H, Moellering DR, Abraham E, Thannickal VJ, et al Pyruvate dehydrogenase kinase 1 participates in macrophage polarization via regulating glucose metabolism. J Immunol. 2015;194(12):6082–9. 10.4049/jimmunol.1402469 25964487 PMC4458459

[45] Curtale G, Rubino M, Locati M. MicroRNAs as Molecular Switches in Macrophage Activation. Front Immunol. 2019;10:799 10.3389/fimmu.2019.00799 31057539 PMC6478758

[46] Tran TH, Krishnan S, Amiji MM. MicroRNA-223 Induced Repolarization of Peritoneal Macrophages Using CD44 Targeting Hyaluronic Acid Nanoparticles for Anti-Inflammatory Effects. PLoS One. 2016;11(5):e0152024 10.1371/journal.pone.0152024 27148749 PMC4858219

[47] Huang C, Liu XJ, Qun Zhou, Xie J, Ma TT, Meng XM, et al MiR-146a modulates macrophage polarization by inhibiting Notch1 pathway in RAW264.7 macrophages. Int Immunopharmacol. 2016;32:46–54. 10.1016/j.intimp.2016.01.009 26800097

[48] Covarrubias AJ, Aksoylar HI, Yu J, Snyder NW, Worth AJ, Iyer SS, et al Akt-mTORC1 signaling regulates Acly to integrate metabolic input to control of macrophage activation. Elife. 2016;5. 10.7554/eLife.11612 PMC4769166 26894960

[49] Huang SC, Smith AM, Everts B, Colonna M, Pearce EL, Schilling JD, et al Metabolic Reprogramming Mediated by the mTORC2-IRF4 Signaling Axis Is Essential for Macrophage Alternative Activation. Immunity. 2016;45(4):817–30. 10.1016/j.immuni.2016.09.016 27760338 PMC5535820

[50] Van den Bossche J, O'Neill LA, Menon D. Macrophage Immunometabolism: Where Are We (Going)? Trends Immunol. 2017;38(6):395–406. 10.1016/j.it.2017.03.001 28396078

[51] Wang F, Zhang S, Vuckovic I, Jeon R, Lerman A, Folmes CD, et al Glycolytic Stimulation Is Not a Requirement for M2 Macrophage Differentiation. Cell Metab. 2018;28(3):463–75 e4. 10.1016/j.cmet.2018.08.012 30184486 PMC6449248

[52] Corcoran SE, O'Neill LA. HIF1alpha and metabolic reprogramming in inflammation. J Clin Invest. 2016;126(10):3699–707. 10.1172/JCI84431 27571407 PMC5096812

[53] Wang T, Liu H, Lian G, Zhang SY, Wang X, Jiang C. HIF1alpha-Induced Glycolysis Metabolism Is Essential to the Activation of Inflammatory Macrophages. Mediators Inflamm. 2017;2017:9029327 10.1155/2017/9029327 29386753 PMC5745720

[54] Li C, Wang Y, Li Y, Yu Q, Jin X, Wang X, et al HIF1alpha-dependent glycolysis promotes macrophage functional activities in protecting against bacterial and fungal infection. Sci Rep. 2018;8(1):3603 10.1038/s41598-018-22039-9 29483608 PMC5827022

[55] Nishi K, Oda T, Takabuchi S, Oda S, Fukuda K, Adachi T, et al LPS induces hypoxia-inducible factor 1 activation in macrophage-differentiated cells in a reactive oxygen species-dependent manner. Antioxid Redox Signal. 2008;10(5):983–95. 10.1089/ars.2007.1825 18199003

[56] Duvel K, Yecies JL, Menon S, Raman P, Lipovsky AI, Souza AL, et al Activation of a metabolic gene regulatory network downstream of mTOR complex 1. Mol Cell. 2010;39(2):171–83. 10.1016/j.molcel.2010.06.022 20670887 PMC2946786

[57] Dweep H, Gretz N. miRWalk2.0: a comprehensive atlas of microRNA-target interactions. Nat Methods. 2015;12(8):697 10.1038/nmeth.3485 26226356

[58] Fleming BD, Chandrasekaran P, Dillon LA, Dalby E, Suresh R, Sarkar A, et al The generation of macrophages with anti-inflammatory activity in the absence of STAT6 signaling. J Leukoc Biol. 2015;98(3):395–407. 10.1189/jlb.2A1114-560R 26048978 PMC4541501

[59] Roy S, Charboneau R, Melnyk D, Barke RA. Interleukin-4 regulates macrophage interleukin-12 protein synthesis through a c-fos mediated mechanism. Surgery. 2000;128(2):219–24. 10.1067/msy.2000.108063 10922995

[60] D'Andrea A, Ma X, Aste-Amezaga M, Paganin C, Trinchieri G. Stimulatory and inhibitory effects of interleukin (IL)-4 and IL-13 on the production of cytokines by human peripheral blood mononuclear cells: priming for IL-12 and tumor necrosis factor alpha production. J Exp Med. 1995;181(2):537–46. 10.1084/jem.181.2.537 7836910 PMC2191875

[61] Brown JM, Recht L, Strober S. The Promise of Targeting Macrophages in Cancer Therapy. Clin Cancer Res. 2017;23(13):3241–50. 10.1158/1078-0432.CCR-16-3122 28341752 PMC5529121

[62] Kowal J, Kornete M, Joyce JA. Re-education of macrophages as a therapeutic strategy in cancer. Immunotherapy. 2019;11(8):677–89. 10.2217/imt-2018-0156 31088236

